# Supplementary material for: HIV preventive practice and its associated factors among street dwellers in Ethiopia: Application of health belief model
Source: PLOS Glob Public Health. 2024 May 3;4(5):e0003199. doi: 10.1371/journal.pgph.0003199 (PMC11068206; doi:10.1371/journal.pgph.0003199)
Supplement: S1 Text — (DOCX) [file pgph.0003199.s001.docx]

**Questionnaires (English Version)**

**PART I: Socio-demographic characteristics and personal information.**

| **S. N** | **Questions** | **Response code** |
| --- | --- | --- |
| 101 | Sex. | 1. Male 2. Female |
| 102 | Age. | _______________years |
| 103 | Religion. | 1. Orthodox Christian 2. Muslim 3. Protestant 4. Catholic 5. Others (if any, specify)________ |
| 104 | Marital status. | 1. Married 2. Single 3. Divorced 4. Widowed |
| 105 | Educational status. | 1. Illiterate 2. Read and write only 3. Primary school (Grades 1-8) 4. Secondary (Grades 9-12) 5. Diploma and above |
| 106 | Do you work to earn money for yourself? | 1. Yes 2. No |
| 107 | If yes for Q106, what do you do to earn money?  [multiple answers are possible] | 1. Begging 2. Shoe shining 3. Carrying 4. Exchange of money for sex 5. Other (if any, specify)------------ |

**Part II: Knowledge related questions**

| **S. N** | **Questions** | **Response code** |
| --- | --- | --- |
| 201 | Can HIV prevented by correct use of condom at every sexual intercourse? | 1. Yes 2. No |
| 202 | Having only one and faithful sexual partner can prevent the risk of getting HIV/AIDS? | 1. Yes 2. No |
| 203 | Can HIV prevented by abstinence? | 1. Yes 2. No |
| 204 | Can HIV transmitted by eating together with HIV infected person? | 1. Yes 2. No |
| 205 | May a healthy looking person have HIV virus in his/her blood? | 1. Yes 2. No |

**Part III: Substance use related questions**

| **S. N** | **Questions** | **Response code** | **s** Skip **kip** | |
| --- | --- | --- | --- | --- |
| **301** | Do you use alcohol? | 1. Yes 2. No | 2🡪 304**ss** | |
| **302** | If Yes for Q 301, How often? | 1. Weekly 2. Two times/week 3. Three times/week 4. More than three times/week 5. Other (if any, specify)------------ | **S** | |
| **303** | Which alcohol do you use?(multiple answer is possible) | 1. Arekie 2. Tella 3. Tej 4. Beer 5. Wayne 6. Other (if any, specify)------------ |  | |
| **304** | Do you use tobacco/ cigarette/? | 1. Yes 2. No | 2🡪 306**ss** | |
| **305** | If Yes for Q 304, How often? | 1. Weekly 2. Two times/week 3. Three times/week 4. More than three times/week 5. Other (if any, specify)------------ |  |  |
| **306** | Do you use Khat? | 1. Yes 2. No | 2🡪 308**ss** |  |
| **307** | If Yes for Q 306, How often? | 1. Weekly 2. Two times/week 3. Three times/week 4. More than three times/week 5. Other (if any, specify)------------ |  |  |
| **308** | Do you use “Ashish”? | 1. Yes 2. No | 2🡪 401**ss** |  |
| **309** | If Yes for Q 308, How often? | 1. Weekly 2. Two times/week 3. Three times/week 4. More than three times/week 5. Other (if any, specify)------------ |  |  |

**Part IV: HIV preventive practice related questions**

| **S. N** | **Questions** | **Response code** | **Skip to** |
| --- | --- | --- | --- |
| 401 | Have you had sexual intercourse after being street dweller? | 1. Yes 2. No |  |
| 402 | Do you use HIV/AIDS preventive practices? | 1. Yes 2. No | 2🡪 501**ss** |
| 403 | Which HIV/AIDS preventive practices do you use? | 1. Abstinence 2. Be faithful (one to one) 3. Condom use 4. Other (if any, specify)------------ |  |

**Part V: Attitude related questions**

| **S. N** | **Questions** | **Strongly disagree (1)** | **Disagree (2)** | **Neutral (3)** | **Agree (4)** | **Strongly Agree (5)** |
| --- | --- | --- | --- | --- | --- | --- |
| 501 | People should Abstain from sex to prevent HIV |  |  |  |  |  |
| 502 | People should be faithful to one partner to prevent HIV |  |  |  |  |  |
| 503 | People should use condom to prevent themselves from HIV |  |  |  |  |  |
| 504 | Caring as an attendant to an HIV positive person cannot transmit HIV |  |  |  |  |  |

**Part VI: Constructs of Health Belief Model items**

**Perceived susceptibility**

| **S. N** | **Questions** | **Strongly disagree (1)** | **Disagree (2)** | **Neutral (3)** | **Agree (4)** | **Strongly Agree (5)** |
| --- | --- | --- | --- | --- | --- | --- |
| 601 | I feel that the chances are high that I can get HIV/AIDS |  |  |  |  |  |
| 602 | I am afraid that I might contract HIV/ AIDS |  |  |  |  |  |
| 603 | I may be exposed to HIV because my partner may be at risk of getting HIV/AIDS |  |  |  |  |  |
| 604 | I might have been infected with HIV/AIDS in some ways |  |  |  |  |  |
| 605 | My sexual behavior is not safe and may be expose me to HIV/AIDS |  |  |  |  |  |

**Perceived severity**

| **S. N** | **Questions** | **Strongly disagree (1)** | **Disagree (2)** | **Neutral (3)** | **Agree (4)** | **Strongly Agree (5)** |
| --- | --- | --- | --- | --- | --- | --- |
| 606 | AIDS causes death |  |  |  |  |  |
| 607 | HIV/AIDS is a disease that could not be cured |  |  |  |  |  |
| 608 | HIV/AIDS is probably the worst disease one can get |  |  |  |  |  |
| 609 | HIV/ADIS causes for stigmatization |  |  |  |  |  |

**Perceived benefit**

| **S. N** | **Questions** | **Strongly disagree (1)** | **Disagree (2)** | **Neutral (3)** | **Agree (4)** | **Strongly Agree (5)** |
| --- | --- | --- | --- | --- | --- | --- |
| 610 | Being abstained from sexual intercourse before marriage is the way one can prevent HIV/AIDS |  |  |  |  |  |
| 611 | Abstinence from sexual intercourse is the first choose to prevent HIV/AIDS |  |  |  |  |  |
| 612 | Being abstained from having sexual intercourse with girl-friend/boy-friend until testing for HIV can prevent its transmission |  |  |  |  |  |
| 613 | Being faithful to my sexual partner will make our love and future life better |  |  |  |  |  |
| 614 | If one can stay with one sexual partner, I belief he/she can prevent transmission of HIV/AIDS |  |  |  |  |  |
| 615 | Consistent condom use can prevent HIV /AIDS transmission |  |  |  |  |  |
| 616 | Consistent condom use prevents transmission of other STI other than HIV/AIDS. |  |  |  |  |  |

**Perceived barrier**

| **S. N** | **Questions** | **Strongly disagree (1)** | **Disagree (2)** | **Neutral (3)** | **Agree (4)** | **Strongly Agree (5)** |
| --- | --- | --- | --- | --- | --- | --- |
| 617 | Peer influence can prevent me from staying abstained from sex |  |  |  |  |  |
| 618 | The street life may affect my being abstained from sexual intercourse |  |  |  |  |  |
| 619 | If I become drunk I may annoyed to have sex with someone who is not my steady sexual partner |  |  |  |  |  |
| 620 | I belief that my friends in this site can pressure me to have sexual intercourse with someone other than my steady sexual partner |  |  |  |  |  |
| 621 | The street site may make me not to stay with one sexual partner |  |  |  |  |  |
| 622 | I belief it is comfortable to use condom during sexual intercourse |  |  |  |  |  |
| 623 | I will be ashamed to buy condom from the  pharmacy or shop |  |  |  |  |  |
| 624 | I don’t know where condom is available |  |  |  |  |  |
| 625 | I haven’t money to buy condom |  |  |  |  |  |
| 626 | I don’t know how condom is utilized |  |  |  |  |  |

**Self-efficacy**

| **S. N** | **Questions** | **Strongly disagree (1)** | **Disagree (2)** | **Neutral (3)** | **Agree (4)** | **Strongly Agree (5)** |
| --- | --- | --- | --- | --- | --- | --- |
| 627 | I am confident I can stay abstained from sex even if I am drunk |  |  |  |  |  |
| 628 | I am confident that I can say no to have sexual intercourse with my boy/girlfriend before marriage even if he/she enforced me to have sex |  |  |  |  |  |
| 629 | I am confident that I can stay abstain even if my friends are have sexual intercourse with someone |  |  |  |  |  |
| 630 | I am confident that I can be with one sexual partner throughout my life |  |  |  |  |  |
| 631 | I am confident that I stay in the street site without sexual inter course |  |  |  |  |  |
| 632 | I am sure that I can control myself from having sexual intercourse other than my steady sexual partner even though I am drunk |  |  |  |  |  |
| 633 | I am confident that I use condom consistently even if I am drunk |  |  |  |  |  |
| 634 | I am confident that I could go to shop and buy condom if I want to get it |  |  |  |  |  |
| 635 | I am confident that I consistently use the condom during my sexual relationship |  |  |  |  |  |

**Cues to action**

| **S. N** | **Questions** | **Strongly disagree (1)** | **Disagree (2)** | **Neutral (3)** | **Agree (4)** | **Strongly Agree (5)** |
| --- | --- | --- | --- | --- | --- | --- |
| 636 | I have heard from media about the importance of abstaining from sex |  |  |  |  |  |
| 637 | My friends were discussing with me to abstain from sex |  |  |  |  |  |
| 638 | My partner discuss with me about the abstinence from sex |  |  |  |  |  |
| 639 | I have heard from media about the importance of having only one sexual partner |  |  |  |  |  |
| 640 | My friends were discussing with me about having only one sexual partner |  |  |  |  |  |
| 641 | My partner discuss with me about having only one sexual partner |  |  |  |  |  |
| 642 | I have heard from media about condom use few days before visiting health institution |  |  |  |  |  |
| 643 | My friends were discussing with me to use condom during sexual intercourse |  |  |  |  |  |
| 644 | My partner talks with me to use condom when to have sexual intercourse |  |  |  |  |  |
