# Supplementary material for: HIV preventive practice and its associated factors among street dwellers in Ethiopia: Application of health belief model
Source: PLOS Glob Public Health. 2024 May 3;4(5):e0003199. doi: 10.1371/journal.pgph.0003199 (PMC11068206; doi:10.1371/journal.pgph.0003199)
Supplement: Attachment — Submitted filename: A Response to Reviewers.docx [file pgph.0003199.s002.docx]

| **Reviewer 1 Comment** | **Authors Response** | **Page No** |
| --- | --- | --- |
| Reviewer #1: "HIV/AIDS Preventive Practice and Its Associated Factors Among Street Dwellers in Ethiopia: Application of Health Belief Model" is a good thesis which may ultimately helps to combat the disease. | Thank you for your kind words |  |
| 1. Street Dwellers should be given an operational definition. | Thank you for your fruitful comments. We have revised it by providing operational definition of street dwellers. | 8 |
| 2. Line number 129-132 "than or equal to 12 years old was included. While street dwellers that were unable to communicate due to severe illness were excluded from the study.  Exclusion criteria: Street dwellers that were unable to communicate due to severe illness were excluded from the study"  First ideas were repeated? Second why those people with severe illness are there in the street and what do you do for them? What type of illness you encountered? | Thank you for your fruitful comments. We would like to notify that we have set exclusion criteria during proposal stage to exclude those with severe illness, but we didn’t encounter any potential participant who are seriously ill. So that this forge us to delete our exclusion criteria since we didn’t get even single study subjects during data collection period. | 7 |
| 3. P= 50% (since there is no previous published research in Ethiopia). ? How? Preventive practice? | Thank you for your fruitful comments. We would like to notify for you that we rephrased it since we tried to show absence of published paper on the specific topic “HIV/AIDS preventive practice and Its associated factors among street dwellers in Ethiopia”. | 8 |
| 4. Line number 297-299 "This discrepancy might be due to differences in partners' involvement in HIV/AIDS preventive practice, demand creation activities, and sample size differences. This idea is Unclear. | Thank you for your fruitful comments. After careful scanning of the referenced study, we decide to remove that because it is pure qualitative study and we cant compare the figure of qualitative and quantitative findings by using frequency. | 17 |
| 5. Line number 304-307, In addition, women's use of HIV/AIDS preventative measures is impacted by a number of factors, including their greater propensity than men to sell sex in order to survive, as well as their vulnerability to sexual exploitation, coercion, and rape. Vast generalization | Thank you for your fruitful comments. After we scan it carefully, we decide to remove those sentence (Line number 304-307) that lead readers for vast generalization. | 17 |
| 6. Rewrite the conclusion | Thank you for your fruitful comments. We have made extensive revision in our conclusion by adding aim of study and implication of the study | 19 |
