# Supplementary material for: HIV preventive practice and its associated factors among street dwellers in Ethiopia: Application of health belief model
Source: PLOS Glob Public Health. 2024 May 3;4(5):e0003199. doi: 10.1371/journal.pgph.0003199 (PMC11068206; doi:10.1371/journal.pgph.0003199)
Supplement: Attachment — Submitted filename: A Response to Reviewers (1).docx [file pgph.0003199.s003.docx]

| **Journal Requirements:** | **Authors Response** | **Page No** |
| --- | --- | --- |
| 1. Please review your reference list to ensure that it is complete and correct. If you have cited papers that have been retracted, please include the rationale for doing so in the manuscript text, or remove these references and replace them with relevant current references. Any changes to the reference list should be mentioned in the rebuttal letter that accompanies your revised manuscript. If you need to cite a retracted article, indicate the article’s retracted status in the References list and also include a citation and full reference for the retraction notice. | Thank you for your fruitful comments. We have reviewed our reference list and we got it complete and correct. | 22 - 25 |
| 2. We have noticed that you have uploaded Supporting Information files, but you have not included a list of legends. Please add a full list of legends for your Supporting Information files after the references list. | Thank you for your fruitful comments. We have added full list of legends for our supporting Information files after the references list. | 29 |
| **Editor Comment** | **Authors Response** | **Page No** |
| Additional Editor Comments (if provided):  Overall, the manuscript has seen significant improvement. However, there is still a need for further refinement in writing quality, particularly in eliminating grammatical errors, typos, and the misuse of punctuation throughout the paper. I have read the abstract and provided some examples and suggestions. However, please note that these are not exhaustive. The authors need to ensure a thorough proofreading of the paper before we could accept it. | Thank you for your fruitful comments. We have made an extensive revision in our manuscript by copy-editing to eliminate grammar errors, typos, and the misuse of punctuation throughout the paper. | All document |
| 1. Follow the structure recommended by PLOSGPH, including a brief background and rationale before stating the objective. | Thank you for your fruitful comments. We have revised it by adding Background section in the abstract | 2 |
| 2. Revise the objective for clarity – "To assess HIV prevention practices and associated factors among street dwellers in Bahir Dar City, Ethiopia using the Health Belief Model." | Thank you for your fruitful comments. We have revised our objective as "To assess HIV prevention practices and associated factors among street dwellers in Bahir Dar City, Ethiopia using the Health Belief Model." | 2 |
| 3. Lines 18-19: "A community-based cross-sectional study was conducted in Bahir Dar City from March 12 to April 30, 2023." | Thank you for your fruitful comments. We have revised lines 18 - 19 based on your comment as “A community-based cross-sectional study was conducted in Bahir Dar City from March 12 to April 30, 2023” | 2 |
| 4. The sentence "Epi-data version 3.1 was used to enter the data, which was then exported to SPSS version for analysis" can be removed. This will provide space for more pertinent information, such as details about key variables and measurements (e.g., how 'HIV prevention practice' was defined and measured). Additionally, the data analysis description can be summarized for conciseness. | Thank you for your fruitful comments. We have made revision by   1. removing the sentence "Epi-data version 3.1 was used to enter the data, which was then exported to SPSS version for analysis" 2. By adding pertinent information, about how 'HIV prevention practice' was defined and measured 3. By writing short summary of data analysis description rather than much detail | 2 |
| 5. Summarize key participants' characteristics at the beginning of the results section. | Thank you for your fruitful comments. We have revised it by adding key participants' characteristics at the beginning of the results section. | 2 |
| 6. Avoid using the term 'HIV/AIDS'; UNAIDS no longer recommends it. The term 'HIV/AIDS prevention' sounds awkward | Thank you for your fruitful comments. We have revised it by avoiding using HIV/AIDS'; UNAIDS. We have used HIV preventive practice rather than “HIV/AIDS prevention' | 2 |
| 7. Line 29: "Education" should not be capitalized. | Thank you for your fruitful comments. We have revised it as "education" | 2 |
| 8. Clarify several variables described in the abstract, such as 'good knowledge,' 'perceived susceptibility,' and 'perceived benefits.' It's important to specify what these variables refer to - knowledge, susceptibility, and benefits of what? | Thank you for your fruitful comments. We have revised it by adding specification for listed variable as “good knowledge about HIV preventive practice, perceived susceptibility for HIV, and perceived benefit of using HIV preventive practice” | 2 |
| 9. Ensure consistency throughout the paper (e.g., lines 29-32: inconsistent use of decimals in CIs). | Thank you for your fruitful comments. We have ensured 3 decimal consistency throughout the paper including CIs and tables finding) | All document |
| 10. In the conclusion, avoid repeating results; instead, summarize key findings supporting recommendations. Make recommendations more specific. | Thank you for your fruitful comments. We have made an extensive revision in our conclusion and we tried to make the recommendations more specific and based on the findings. | 2 |
| These revisions will enhance the clarity and coherence of the manuscript. | Thank you for your fruitful comments. We have made an extensive revision in our manuscript to enhance the clarity and coherence of the manuscript | All document |
